# Lianweng Granules Alleviate Intestinal Barrier Damage via the IL-6/STAT3/PI3K/AKT Signaling Pathway with Dampness-Heat Syndrome Diarrhea

**DOI:** 10.3390/antiox13060661

**Published:** 2024-05-28

**Authors:** Jianyu Lv, Yuchen Fu, Yu Ga, Chao Han, Yimeng Fan, Yuanyuan Wei, Sijia Hao, Zhihui Hao

**Affiliations:** 1 Innovation Centre of Chinese Veterinary Medicine, College of Veterinary Medicine, China Agricultural University, Beijing 100193, China; s20223050978@cau.edu.cn (J.L.); s20233051040@cau.edu.cn (Y.F.); s20203050825@cau.edu.cn (Y.G.); s20233050986@cau.edu.cn (C.H.); b20223050425@cau.edu.cn (Y.F.); b20203050389@cau.edu.cn (Y.W.); 2 Key Biology Laboratory of Chinese Veterinary Medicine, Ministry of Agriculture and Rural Affairs, Beijing 100193, China; 3 National Center of Technology Innovation for Medicinal Function of Food, National Food and Strategic Reserves Administration, Beijing 100193, China; 4 College of Traditional Chinese Medicine, Inner Mongolia Medical University, Hohhot 010059, China; s20223050977@cau.edu.cn

**Keywords:** network pharmacology, dampness-heat syndrome diarrhea, serum pharmacochemistry, IL-6/STAT3/PI3K/AKT

## Abstract

Dampness-heat syndrome diarrhea (DHSD) is a common clinical disease with a high prevalence but still has no satisfactory therapeutic medicine, so the search for a safe and effective drug candidate is ongoing. This study aims to explore the efficacy and mechanisms of Lianweng granules (LWG) in the treatment of DHSD and to identify the blood transport components of LWG. We assessed the efficacy of LWG in DHSD by various in vivo metrics such as body weight, disease activity index (DAI), histopathologic examination, intestinal barrier function, levels of inflammatory, apoptotic biomarkers, and oxidative stress. We identified the blood components of LWG using ultra-high performance liquid chromatography-mass spectrometry/mass spectrometry (UHPLC-MS/MS), and the resolved key components were used to explore the relevant targets. We next predicted the potential mechanisms of LWG in treating DHSD using network pharmacology and molecular docking based on the relevant targets. Finally, the mechanisms were validated in vivo using RT-qPCR, Western blotting, ELISA, and immunofluorescence and evaluated in vitro using Cell Counting Kit-8 (CCK-8), small interfering RNA, cellular enthusiasm transfer assay (CETSA), and drug affinity response target stability (DARTS). Ninety-one pharmacodynamic components of LWG enter the bloodstream and exert possible therapeutic effects. In vivo, LWG treatment improved body weight, reduced colonic injury and DAI scores, lowered inflammation, oxidative stress, and apoptosis markers, and partially restored intestinal barrier function in DHSD mice. Guided by network pharmacology and molecular docking, it is suggested that LWG may exert therapeutic effects by inhibiting IL-6/STAT3/PI3K/AKT signaling. LWG significantly decreased the expression of IL-6, p-STAT3, p-PI3K, p-AKT, and other proteins. These findings were supported by in vitro experiments, where CETSA, DARTS, and siRNA evidenced LWG’s targeting of STAT3. LWG targeted STAT3 to inhibit inflammation, oxidative stress, and apoptosis in the colon, thereby restoring the intestinal barrier function to some extent and exerting a therapeutic effect on DHSD.

## 1. Introduction

One of the most common clinical diseases, diarrhea often causes abdominal pain, vomiting, intestinal bleeding, and other symptoms in patients, which may even lead to systemic symptoms or cancer in severe cases [1]. Morbidity and mortality from diarrhea are increasing every year and pose a serious public health problem for most developing countries [2]. DHSD is a common type of diarrhea under the theory of traditional Chinese medicine. A survey of Chinese medicine evidence on diarrhea showed that DHSD is the most common type of diarrhea (57.63%), is commonly found in tropical and subtropical regions, and is caused by high temperature and high humidity environments and inappropriate dietary habits [3]. Its main symptoms include diarrhea and abdominal pain, fever, mucosal damage, intestinal inflammation, and even systemic inflammation [4]. Even worse, it can develop into cancer if not treated properly. Conventional treatments, including corticosteroids, antibiotics, and immunosuppressants, often fall short or pose risks due to adverse effects [5]. Fortunately, traditional medicines are uniquely suited to treating diarrhea. Currently, the World Health Organization, in collaboration with other international organizations, has begun to establish diarrhoeal disease control programs globally, focusing on the use of traditional medicines for treatment [4]. Among them, traditional Chinese medicine (TCM) is one of the most important traditional medicines in the world, which has shown good application prospects in the treatment of diarrhea diseases.

Currently, TCM has been widely used to treat gastrointestinal diseases [6], but the unknown material basis and pathways of action have seriously hindered its internationalization [7]. Serum pharmacochemistry aims to screen the efficacious substance matrix of TCM from the components absorbed into the blood after oral administration, reflecting the multi-component synergy for overall biological effects and has gained wide acceptance and application [8], but it does not involve the study of functional mechanisms, while network pharmacology can make up for the lack of serum pharmacochemistry by constructing complex biological networks in the organism and predicting the functional mechanisms during drug therapy [9].

There is a mountain of evidence that suggests that repairing the damaged intestinal epithelial barrier and restoring its function may be the key to treating intestinal disease, and the most common factor leading to intestinal barrier damage is intestinal inflammation [10]. A flare-up of inflammatory factors in the gut is a typical feature of DHSD, especially IL-6 [11]. Previous studies have shown that binding of IL-6 to its membrane-bound receptor can lead to the aberrant activation of signal transducer and activator of transcription-3 (STAT3) [12]. Phosphorylated STAT3 enters the nucleus as a dimerized, participates in gene transcription, and further contributes to the development of cytokine storms that ultimately jeopardize the whole body [13]. Phosphorylated STAT3 can also exacerbate barrier damage in the colon by promoting the differentiation of Th17 cells [14]. On the other hand, STAT3 acts as an upstream node of Phosphoinositide 3-Kinase (PI3K), and aberrant activation of STAT3 also affects PI3K/AKT signaling [15]. In patients with p-STAT3 overexpression, most of them also overexpressed p-AKT, both of which play a key role in the regulation of apoptosis [16]. Numerous studies have shown that upregulation of PI3K/AKT signaling in the intestine often predicts the rise of apoptosis and oxidative stress, which further leads to damage to the intestinal barrier and induces exacerbation of the disease [17]. The development of targeted drugs for STAT3 provides new perspectives in the clinical fight against intestinal diseases [18].

LWG is a traditional Chinese medicine formula composed of *coptidis rhizoma* (huanglian in Chinese, HL), *pulsatilliae radix* (baitouweng in Chinese, BTW), *rhubarb charcoal* (dahuangtan in Chinese, DHT), and *geranium wilfordii maxim* (laoguancao in Chinese, LGC). These herbs have been reported to have anti-inflammatory and antioxidant properties, which are potential candidates for the treatment of intestinal disorders [19,20,21,22]. Our previous study showed that LWG has significant therapeutic effects on DHSD, but the potential mechanism is unknown. Integration of network pharmacology and serum pharmacochemistry may be a potential strategy to study the pharmacological mechanisms of LWG on DHSD.

In our study, we combined serum medicinal chemistry, network pharmacology, and in vivo and in vitro experiments to reveal that LWG exerts its therapeutic effects on DHSD through inhibition of the IL-6/STAT3/PI3K/AKT signaling pathway and to identify the targeted binding of LWG to STAT3. This study provides evidence to unravel the pathogenesis of DHSD and offers a safe and effective drug candidate.

## 2. Materials and Methods

### 2.1. Medicine

First, 400 g of LWG (Qingdao Kangdien Pharmaceutical Co., Ltd., Qingdao, China) was dissolved in 1 L of distilled water to prepare a stable solution of 0.4 g/mL. Weighing 120 g of HL and BTW and 80 g of DHT and LGC (Tongrentang Pharmacy, Beijing, China) according to the formulation ratio, the above herbs were divided into four containers, soaked fully in 2 L of pure water, boiled for 50 min, filtered and concentrated so that the volume of the solution finally reached 1 L. The concentrated solutions of the four herbs were collected. All the above medicines were placed in a refrigerator at 4 °C for use. The quality of LWG was characterized using HPLC, and all the experimental procedures, as well as the results, are shown in the Supplementary File. Based on the interspecies dose conversion formula, the administered dose for mice was calculated to be 1 g/kg/day.

### 2.2. Collection of LWG-Containing Serum and UHPLC-MS/MS Analysis

The mice were purchased from SiPeiFu Biotechnology Co., Ltd. (Beijing, China) under license number (SCXK (Jing) 2019-0010). All SPF-grade male BALB/c mice (6–8 weeks old) for this study were housed in specific pathogen-free facilities at 25 ± 1 °C with a standard 12-h light/12-h dark cycle, and mice were provided with water and food that were available ad libitum. All animal procedures were approved by the Animal Care Committee of the Chinese Agricultural University. The experimental protocols were approved by the Institutional Animal Care and Use Committee of China Agricultural University (No. Aw91903202-2-1) on 19 September 2023.

A total of 36 were divided into a control group (*n* = 6, equal volume of saline), LWG group (*n* = 6, 1 g/kg of LWG dissolved solution), BTW group (*n* = 6, 0.3 g/kg of BTW concentrate), HL group (*n* = 6, 0.3 g/kg of HL concentrate), DHT group (*n* = 6, 0.2 g/kg of DHT concentrate), and LGC group (*n* = 6, 0.2 g/kg of LGC concentrate). Mice were administered continuously for 7 days and all mice were sacrificed 60 min after the last dose was given. Mice were injected with 1% pentobarbital anesthetic before sacrifice, and blood was collected from the abdominal aorta for the preparation of serum samples. The serum samples were characterized using UHPLC-MS/MS, and detailed conditions for mass spectrometry analysis are shown in the Supplementary File.

### 2.3. Evaluation of Differential Components

Differential components entering the blood were screened for with mass spectrometry identification of control and LWG groups. The differential components were evaluated using the online tool Swiss ADME (http://www.swissadme.ch/, accessed on 11 April 2023) based on the Lipinski principle. In short, hydrogen bond donors (Hdon) and hydrogen bond acceptors (Hacc) ≤ 5; the number of rotatable bonds (Rbon) ≤ 10; the lipid-water partition coefficient (MLOGP) ≤ 4.15; and the relative molecular mass (M/W) ≤ 500. In addition, we conducted further screening with the topological polar surface area (TPSA) ≤ 100. The differential components meeting the above criteria were considered to have significant pharmacological activity and could be used for further systematic pharmacological analysis.

To exclude extraneous environmental factors and contamination of reagents during sample collection, the above results were traced based on the results of the DHT, HL, BTW, and LGC groups. The components traced from the four herbs were considered potential therapeutic substances for the LWG against DHSD. The results were uploaded to Cytoscape 3.9.1 software (https://cytoscape.org/, accessed on 19 April 2023) for visualization. Detailed information on the traced ingredients is shown in Table 1.

### 2.4. Network Pharmacology Analysis

#### 2.4.1. Collection of Relevant Targets for LWG and DHSD

The targets related to the above potential therapeutic substances were collected using the online tool Swiss Target Prediction (http://www.swisstargetprediction.ch/, accessed on 14 May 2023), and the screening criteria were “Probability > 0”. DHSD-related targets were collected from the GeneCards Database (https://www.genecards.org/, accessed on 15 May 2023), Online Mendelian Inheritance in Man (OMIM, https://www.omim.org/, accessed on 15 May 2023), Therapeutic Target Database (TTD, http://db.idrblab.net/ttd/, accessed on 15 May 2023), and the GeneCards database was selected only for targets with a “score” > 1. The targets associated with LWG and DHSD were uploaded to the online tool Venny 2.1.0 (https://bioinfogp.cnb.csic.es/tools/venny/, accessed on 19 May 2023) to obtain crossover targets as potential targets of action.

#### 2.4.2. Construction of Protein–Protein Interaction Network (PPI)

The intersecting targets were uploaded to the STRING database (https://cn.string-db.org/, accessed on 19 May 2023) to build the PPI network, set the highest confidence level (confidence level > 0.9), and submit the obtained results to Cytoscape 3.9.1 software (https://cytoscape.org/, accessed on 25 May 2023). The Analyze Network plug-in was used to systematically analyze the network parameters. The MCC (maximum cluster centrality) algorithm in the cytoHubba plug-in was used to score all nodes in the complex network; the top 30 nodes were selected as the core targets, and the interaction relationships between the core targets were visualized.

#### 2.4.3. Construction of “DHSD-LWG-Compounds-Core Targets”

Cytoscape 3.9.1 software (https://cytoscape.org/, accessed on 29 May 2023) was used for constructing the “DHSD–LWG–compounds-core targets” network with the core genes and serum transition components. The network was systematically analyzed using the Analyze Network plug-in, which calculates the degree value of each node and focuses on the nodes with higher degree values.

#### 2.4.4. Enrichment Analysis

To further investigate the potential mechanism of action, bioinformatics analysis was implemented in this study using the DAVID database (https://david.ncifcrf.gov/, accessed on 11 June 2023), mainly including Gene ontology (GO) and Kyoto Encyclopedia of Genes and Genomes (KEGG) enrichment analysis, which included Biological Process (BP), Molecular Function (MF), Cellular Component (CC) for GO, and all bioinformatics data analysis was performed on the Bioinformatics website (http://www.bioinformatics.com.cn/, accessed on 15 June 2023).

### 2.5. Molecular Docking

The target genes of STAT3, PI3K, IL-6, and AKT were searched in the PDB database (https://www.rcsb.org, accessed on 17 June 2023), of which the 3D protein conformations with a crystal resolution of lower than 2.5 Å as determined by X crystal diffraction were acquired. The Mol2 format files of LWG key active ingredients were downloaded from the TCMSP platform (https://old.tcmsp-e.com/tcmsp.php, accessed on 18 June 2023). The docking results were obtained by running autogrid4 and autodock4, by which the binding energies were revealed. The partial diagram of molecular docking was then generated using the PyMol software (v.2.5.1, https://pymol.org/, accessed on 29 June 2023).

### 2.6. Animal Model

Mice were randomized into 5 groups: the control group (*n* = 9), the DHSD group (*n* = 9), the DHSD + sulfasalazine treatment group (100 mg/kg, *n* = 9), and the DHSD + LWG treatment group (low and high dosage of 0.5 and 1 g/kg, *n* = 9). Except for the control group, mice in the other groups were fed 30% honey water and lard (0.4 mL/20 g every other day) for 10 consecutive days, white wine (56% ethanol, 0.2 mL/20 g) for 5 consecutive days at 33 ± 2 °C and 93% ± 2% humidity, and intraperitoneally injected with *Escherichia coli* (bacterial no. CVCC231; 1.06 × 10^8^ CFU/mL; 0.2 mL/20 g), the mice in control group were fed a standard diet and injected intraperitoneally with an equal volume of saline throughout [4]. After the successful establishment of the DHSD mice model, mice in the DHSD + sulfasalazine treatment group and the DHSD + LWG treatment group were treated by gavage with LWG and sulfasalazine once daily for seven days. In the control and DHSD groups, mice were gavaged with an equal volume of normal saline. All mice were fed with a standard diet in natural conditions. Mice were euthanized with 1% pentobarbital on day 25, and blood was collected through the abdominal aorta. The tissue samples were collected for molecular analyses.

### 2.7. Cell Culture

IEC-6 cells were purchased from the Cell Bank of the Chinese Academy of Sciences (Shanghai, China). The cells were cultured in a DMEM culture medium containing 10% fetal bovine serum and incubated at 37 °C in 5% CO_2_.

Considerable evidence suggests that LPS can cause oxidative stress as well as inflammation in IEC-6 cells and inhibit intercellular tight junctions with significant cellular damage, so LPS was chosen as an inducer for modeling in this study. IEC-6 cells were inoculated in 6-well plates and divided into control, model, (+)magnoflorine, berberine, quillaic acid, and LWG groups. The (+)magnoflorine, berberine, quillaic acid, and LWG groups were stimulated with LPS (1 μg/mL) for 24 h and treated with drug or drug-containing serum, respectively. The control group did not receive any treatment, while the model group received only LPS (1 μg/mL) stimulation. The LWG group was treated with the appropriate concentration of drug-containing serum, and the remaining subgroups were treated with the corresponding drugs, respectively.

### 2.8. Cell Viability Assay and Cell Transfection

IEC-6 cells (5 × 10^4^/mL) were inoculated into 96-well plates and incubated with LWG-containing serum or drug for 24 h. Then, 10 μL of CCK-8 (Dojindo, Kumamoto, Japan) was added to the plate, and the absorbance was measured at 450 nm after 2 h of incubation.

IEC-6 cells were transfected with STAT3-specific small interfering RNA (SiSTAT3, Genomeditech, Shanghai, China) using Lipofectamine 3000 (Thermo Fisher Scientific, Waltham, MA, USA) according to the manufacturer’s instructions. SiSTAT3 was deliquated to an ultimate concentration of 50 nM. Then, 24 h after transfection, the validity of STAT3 knockdown was confirmed. Primer sequences for SiSTAT3 are available in the Supplementary File.

### 2.9. Determination of ROS, CAT, and SOD Levels

Catalase (CAT) and superoxide dismutase (SOD) activities were determined using commercial kits (Nanjing Jiancheng Bioengineering Institute, Nanjing, China), and all experimental steps were carried out according to the protocols provided in the kits.

Cells were seeded in a 6-well plate (10^5^ cells/well) and allowed to adhere overnight. After the incubation, the seeded cells were incubated with 1 mL of 10 µM 2′,7′-Dichlorodihydrofluorescein diacetate (DCFH-DA, Solarbio, Beijing, China) in culturing media for 1 h. Upon completion, the cells were washed with phosphate buffer saline (PBS) twice, followed by treatments with selected compounds at different concentrations for 24 h. Then, the treated cells were harvested, washed, and re-suspended in PBS, followed by flow-cytometric analysis at the Fluorescein 5-isothiocyanate (FITC) channel.

### 2.10. Evaluation of DAI and Histopathological Observations

In the DHSD animal model, the severity of the disease was assessed using the DAI scoring system, which consists of three items, including weight loss, stool consistency, and blood in the stool; the scoring criteria of the DAI scoring are shown in Table S1 [23]. The severity of the disease was positively correlated with the sum of the scores of the above three items.

For histological analysis of the colon, the colon was fixed in 10% neutral formalin buffer and subsequently embedded in paraffin, which was cut into 5 μm slices and stained using hematoxylin and eosin (H&E) and Alcian blue-periodic acid-Schiff (AB-PAS).

### 2.11. Biochemical Analysis

The serum or cell lysate was tested for cytokine using an Elisa kit (Beyotime, Shanghai, China). The procedure was carried out according to the instructions of the kit, and the absorbance at 450 nm was measured using a microplate reader. Targets include IL-6, IL-1β, TNF-α, and Caspase3.

### 2.12. Western Blot

Total protein was extracted using a total protein extraction kit and protein concentrations were determined with a bicinchoninic acid assay kit. The sodium dodecyl sulfate–polyacrylamide gel electrophoresis (SDS-PAGE) was used to separate proteins and incubated with primary antibodies with shaking overnight at 4 °C. These secondary antibodies were HRP-conjugated anti-rabbit secondary antibodies (1:10,000, Beijing Biosynthesis Biotechnology, Wuhan, China). Bands were visualized by a gel imaging analysis system (Bio-Rad, Hercules, CA, USA) after enhanced chemiluminescence (ECL) color rendering. β-actin served as an internal control when appropriate. All antibody information is shown in detail in Table S3 of the Supplementary File.

### 2.13. RT-qPCR

Total RNA was extracted with Trizol, trichloromethane, and isopropyl alcohol in an RNase-free environment. A NanoDrop 2000 spectrophotometer (Thermo Fisher Scientific, Waltham, MA, USA) was used to determine RNA concentrations and purity at 260 nm and 280 nm. RNA was reverse-transcribed into complementary DNA (cDNA) using a commercial kit (Thermo Fisher Scientific, Waltham, MA, USA) and a T100 (Bio-Rad, Hercules, CA, USA) thermocycler. The mRNA levels in the colon were detected by GoScript Reverse Transcription System (Promega Biotech, Beijing, China) on a CFX 96 real-time system instrument (Bio-Rad, Hercules, CA, USA) with the following amplification program: 95 °C 120 s and 45 cycles of 95 °C for 5 s, 64 °C for 30 s and 72 °C for 30 s using specific primers (Table S2) [24,25,26,27,28,29,30]. The housekeeping gene *beta-actin* was used to standardize the gene expression, and the relative expression level of each gene was calculated using the 2^−ΔΔCT^ method [31].

### 2.14. Transmission Electron Microscopy (TEM) Study and TdT-Mediated dUTP-Biotin Nick End Labeling (TUNEL) Staining

After fixation of the colon in 2% glutaraldehyde for 6 h (4 °C), it was dehydrated, embedded, sectioned, and stained. Sections were examined according to standard procedures and evaluated using TEM (JEM1230, JEOL, Tokyo, Japan) observation.

To detect apoptosis in colonic epithelial cells, colon tissue sections were subjected to TUNEL staining (Wuhan Boster Biological Technology, Wuhan, China). The staining results were observed using a Y-TV55 fluorescence microscope (Nikon, Tokyo, Japan).

### 2.15. Immunofluorescence

The slices were washed three times with phosphate-buffered saline with tween 20 (PBST) for 5 min and blocked with 5% bovine serum albumin for 30 min. The samples were incubated with anti-tight junction protein ZO-1 (ZO-1) antibody as well as anti-Claudin1 antibody at 4 °C overnight and washed and incubated with goat anti-rabbit IgG H&L chains (Cell Signaling Technology, Danvers, MA, USA). The nuclei were stained with 4′,6-diamidino-2-phenylindole (DAPI, Beyotime, Shanghai, China) and then washed with PBS and sealed with glycerol. The number of positive cells was observed using fluorescence microscopy (Olympus, Tokyo, Japan).

### 2.16. CETSA

Normal cultured cells were exposed to serum with or without the drug, and after 12 h, the cells were suspended in PBS (with added protease and phosphatase inhibitors), dispensed in separate containers, and heated at the indicated temperatures for 3 min. Cells were lysed three times using a liquid nitrogen cycle, followed by centrifugation at 4 °C for 20 min at 120,000 rpm, and the supernatant was separated for Western blot analysis. An anti-STAT3 antibody was provided by HuaBio (Hangzhou, China).

### 2.17. DARTS

IEC-6 cells were co-incubated with LPS for 24 h. Cell lysates were collected (without the addition of protease inhibitors) and normalized for protein concentration using the Bicinchoninic Acid Assay (BCA, Thermo Fisher Scientific, Waltham, MA, USA) kit. Drug-containing serum was added to the cell lysate and incubated for 0.5 h. Subsequently, different ratios of proteases were added and incubated again for 0.5 h. The cell lysates were then incubated for 0.5 h with the protease inhibitor. Finally, protease inhibitors were added and mixed with SDS-PAGE upsampling buffer and heated at 100 °C for 10 min for Western blot experiments.

### 2.18. Statistical Analysis

Statistical analyses were performed using SPSS 21.0 (IBM, Chicago, IL, USA). One-way ANOVA was performed after the comparison of variance homogeneity tests among multiple groups. When the variance was not uniform, two independent sample *t*-tests were used. The difference was significant at *p* < 0.05, and the difference at *p* < 0.01 was extremely significant.

## 3. Result

### 3.1. Identification of Blood Components and Network Pharmacology Predicts Key Targets

**Figure 1:**
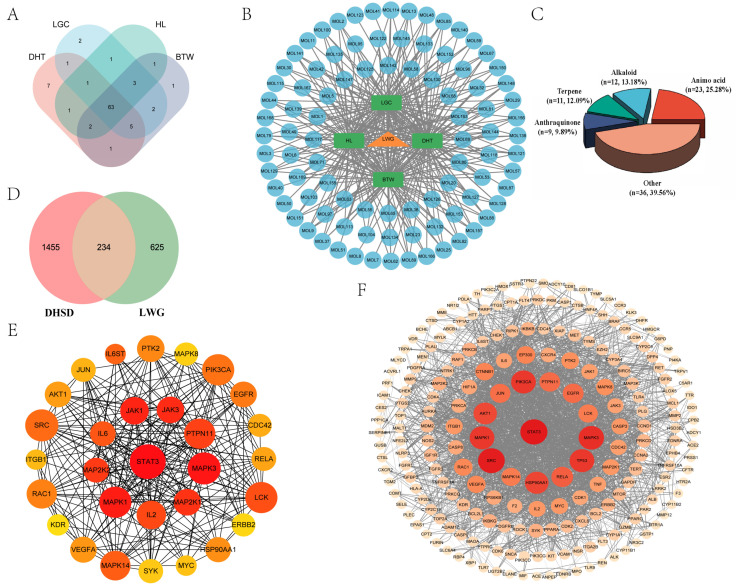
Analysis of prototype drug components and key target analysis of LWG. (**A**) Intersection of four herbal blood components. (**B**) Network analysis of LWG–herbs–prototypical components, the yellow triangular nodes represent the herbal formula particles LWG, the green rectangular nodes represent the four herbs, and the blue circular nodes represent the prototypical drug ingredients. HL: coptidis rhizoma, BTW: pulsatilliae radix, DHT: rhubarb charcoal, LGC: geranium wilfordii maxim. (**C**) Classification of prototypical drug components. (**D**) Target intersection of drug prototype components with DHSD (**E**) PPI analysis network graph. (**F**) Key target collection in PPI network graph. Node size and color in the network graph are positively correlated with the degree value. Redder colors and larger nodes represent larger degree value.

The results of Figure S1 demonstrate that the LWG used in this study satisfied the quality standards. The data of each group were annotated according to the results of UHPLC-MS/MS (Figure S2). A total of 218 blood-intake differential components were screened based on the results of the LWG group (Figure S2B) and the control group (Figure S2A), and 169 potentially pharmacophoric components were finally identified after Lipinski principle screening. After the above components were subjected to herbal traceability, a total of 91 drug prototype components were identified (Table 1), 91 drug prototype ingredients were traced, and the results were visualized as a network structure, with the connecting lines between the nodes representing the compound sources (Figure 1B). These components were categorized as amino acid (23 species, 22.77%), alkaloid (12 species, 11.88%), terpene (11 species, 10.89%), anthraquinone (9 species, 8.91%), etc. (Figure 1C). Regional assignments based on the source of the compounds revealed 63 identical blood-entry components in the four herbs (Figure 1A), showing striking similarities.

Based on 91 herbal archetypal ingredients, a total of 859 LWG-related targets were obtained in the SwissTargetPredicition database, and a total of 1689 DHSD-related targets were obtained in the GeneCards, OMIM, and TTD databases. A total of 234 overlapping targets were finally identified by integrating LWG- and DHSD-related targets (Figure 1D). Next, we used the STRING database to build a protein–protein interaction network graph, which consisted of 227 nodes and 2156 interaction links (Figure 1E). Thirty core targets were screened according to the MCC algorithm in Cytoscape 3.9.1 software, and the top-ranked targets were STAT3, JAK1, JAK3, MAPK3, MAPK1, and IL-6 (Figure 1F).

### 3.2. The Enrichment Results Showed That the PI3K/AKT Pathway Was Involved in the Pharmacological Mechanism of LWG

**Figure 2:**
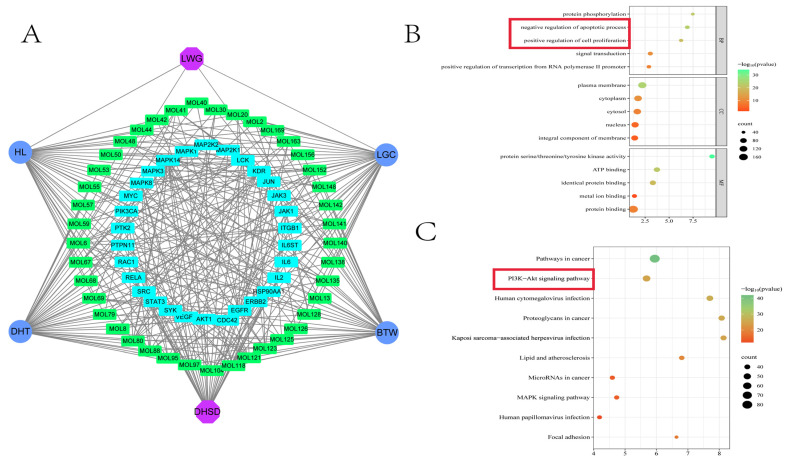
Network Pharmacology Analysis of LWG Components with DHSD. (**A**) Network diagram for “DHSD–LWG–drug prototype component–core targets”. The dark blue nodes represent the four herbs, the green nodes represent the drug prototype components, and the light blue nodes represent the core targets. (**B**) GO enrichment analysis. (**C**) KEGG enrichment analysis. Red boxes represent biological processes that may play a key role in this study.

In order to analyze the relationship between compounds, diseases, and core targets as a whole, 91 compounds were mapped to 30 core targets, and a “DHSD–LWG–drug prototype components–core targets” network graph was obtained, which consisted of 78 nodes and 462 interaction lines (Figure 2A), indicating that the intervention of LWG on DHSD is a complex multi-component, multi-target synergistic action process. Among them, the drug prototype components with higher degree values were MOL80 ((+)magnoflorine), MOL121 (berberine), and MOL6 (quillaic acid).

GO enrichment results showed that during the treatment of DHSD by LWG, BP mainly included protein phosphorylation, negative regulation of apoptotic process, positive regulation of cell proliferation, signal transduction, and positive regulation of transcription from RNA polymerase II promoter; MF mainly included protein serine/threonine/tyrosine kinase activity, ATP binding, identical protein binding, metal ion binding, and protein binding; CC mainly included plasma membrane, cytoplasm, cytosol, nucleus, integral component of membrane (Figure 2B). The enrichment results of the KEGG pathway indicated that the potential mechanism of action of LWG on DHSD may involve pathways in cancer, PI3K-Akt signaling pathway (Figure 2C).

### 3.3. Molecular Docking

**Figure 3:**
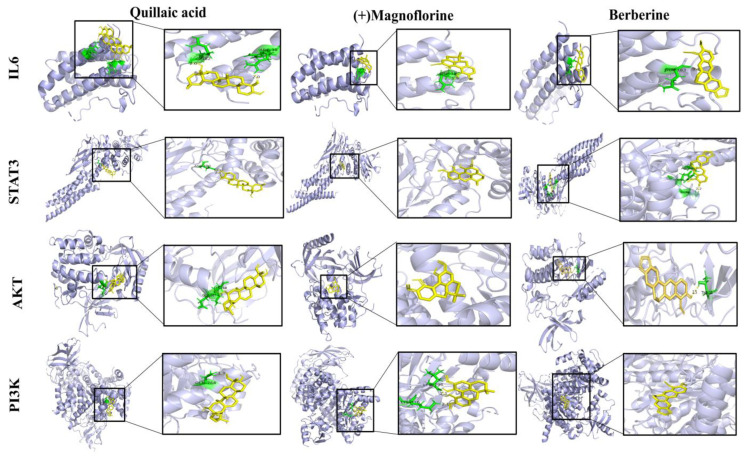
Molecular docking patterns of drug prototype components and targets. The yellow dashed lines represent hydrogen bonding forces. The yellow color represents the ligand and the green color represents the amino acid residues to which the compound binds to the protein.

This study predicted the affinity between the drug prototype components and the target using the molecular docking method. The results showed that the binding energies of both the prototype components and the targets were less than −3 kcal/mol, which indicated that the two had lower binding energies and could form a more stable conformation (Figure 3). Remarkably, STAT3 produced hydrogen-bonding interactions with all three key compounds, showing striking affinity.

### 3.4. LWG Restores Intestinal Morphology

**Figure 4:**
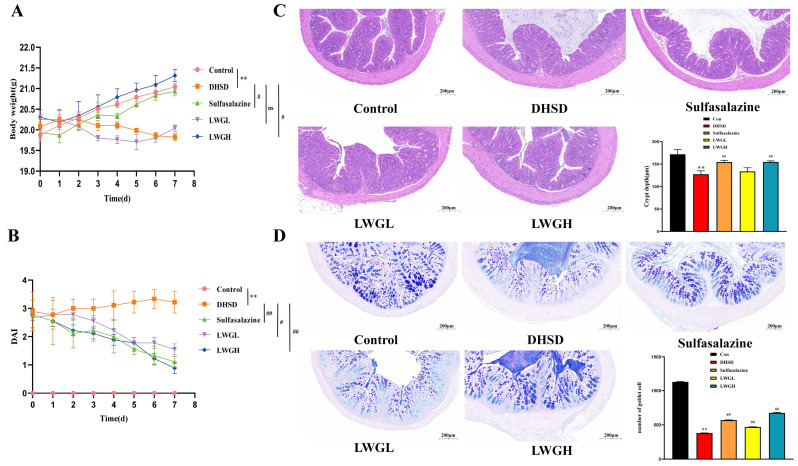
LWG ameliorates diarrhea and intestinal injury in the DHSD model. (**A**) A record of weight changes. (**B**) Analysis of the disease activity index (DAI). (**C**) H&E staining of the colon and depth of the crypts. (**D**) AB/PAS staining of the colon and the number of goblet cells. Statistical comparisons were made with the control group, denoted as ** *p* < 0.01. Comparisons with the DHSD group are denoted as ^#^ *p* < 0.05, ^##^ *p* < 0.01.

We investigated whether LWG could treat the DHSD mouse model induced by complex factors. As shown in Figure 4A, the body weight of the DHSD model mice was significantly lower than that of the control group (*p* < 0.01), whereas the high dose of LWG and sulfasalazine ameliorated the loss of body weight caused by the DHSD model (*p* < 0.05). Calculation of DAI scores of mice in each group showed that LWG and sulfasalazine alleviated parameters such as diarrhea and blood in the stool (Figure 4B). H&E staining showed that LWG reversed tissue damage with inflammatory cell infiltration and increased crypt depth in DHSD mice (Figure 4C). Quantification of the number of goblet cells using AB and APS staining showed that the number of goblet cells was significantly reduced in DHSD mice compared to control mice, and LWG treatment attenuated this effect (*p* < 0.05; Figure 4D).

### 3.5. LWG Alleviates Inflammation, Oxidative Stress, and Apoptosis

**Figure 5:**
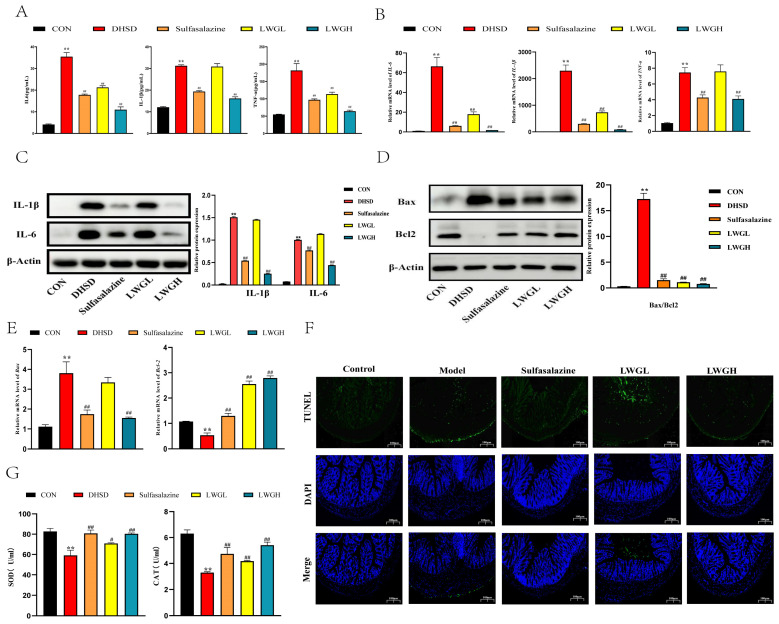
LWG ameliorates inflammation and apoptosis in the DHSD Model. (**A**) Effect of LWG on pro-inflammatory cytokines in the serum of DHSD model mice, including IL-6, IL-1β, and TNF-α. (**B**) Relative mRNA expression of *IL-6*, *IL-1β*, and *TNF-α*. (**C**,**D**) Relative protein levels of IL-6, IL-1β, TNF-α, Bax, and Bcl-2. (**E**) Relative mRNA expression of *Bax* and *Bcl-2*. (**F**) Representative images of TUNEL staining of the colon. (**G**) The level of oxidative stress biomarker CAT and SOD. Statistical comparisons were made with the control group, denoted as ** *p* < 0.01. Comparisons with the DHSD group are denoted as ^#^ *p* < 0.05, ^##^ *p* < 0.01.

Inflammation, oxidative stress, and apoptosis in the colon may be the key to the impaired intestinal barrier. We observed that IL-6, TNF-α, and IL-1β in the serum of DHSD mice were significantly increased (*p* < 0.01) but decreased after treatment with LWG (*p* < 0.01; Figure 5A). In addition, examination of the results of WB and RT-qPCR showed that LWG intervention significantly reduced the levels of inflammatory factors in the colon (*p* < 0.01), and the effect of LWGH appeared to be superior to that of sulfasalazine (Figure 5B,C). On the other hand, results showed that LWG treatment with mice in the DHSD group increased the expression of anti-apoptotic protein (Bcl 2) and decreased the levels of pro-apoptotic proteins (Bax) in the colon (Figure 5D,E). As verified by TUNEL staining, it was found that the apoptosis rate of intestinal epithelial cells in DHSD group mice was significantly increased when compared with the control group (*p* < 0.01), and LWG treatment significantly improved this situation (*p* < 0.01, Figure 5F). In addition, we observed a significant decrease in the activity of antioxidant enzymes (SOD and CAT) in the intestine of DHSD model mice, a phenomenon that was restored after LWG treatment (*p* < 0.01, Figure 5G). In conclusion, the findings suggest that LWG attenuates inflammation, oxidative stress, and apoptosis in the colon of DHSD mice.

### 3.6. LWG Repairs Barrier Function and Inhibits STAT3/PI3K/AKT Activation in DHSD Mice

**Figure 6:**
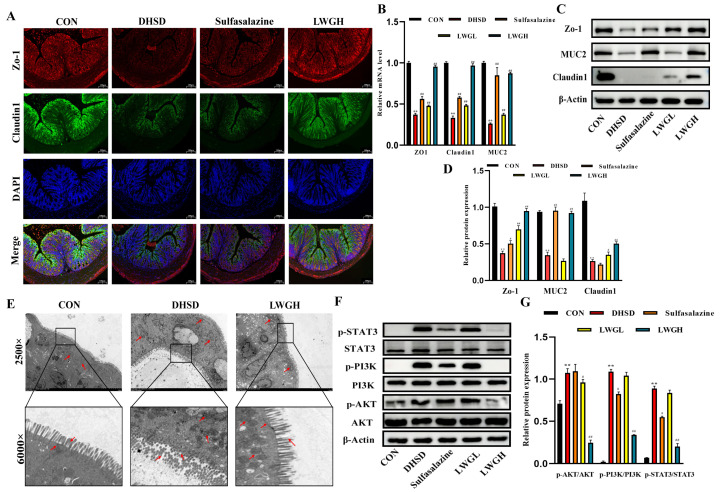
LWG repairs barrier function and inhibits STAT3/PI3K/AKT activation in DHSD mice. (**A**) Representative images of fluorescent staining of ZO-1 (red) and Claudin1 (green) in the colon (10×). (**B**) Relative mRNA expression of *Zo-1*, *Claudin1*, and *MUC2*. (**C**,**D**) Relative protein levels of Zo-1, MUC2, and Claudin1. (**E**) Ultrastructural features of mouse colon captured by TEM. (**F**,**G**) Relative protein levels of p-STAT3/STAT3, p-PI3K/PI3K, and p-AKT/AKT. Statistical comparisons were made with the control group, denoted as ** *p* < 0.01. Comparisons with the DHSD group are denoted as ^#^ *p* < 0.05, ^##^ *p* < 0.01. The red arrows represent noteworthy sites in the ultrastructural pictures of different groupings of colons.

By immunofluorescence analysis of tissue sections of the mouse colon, the fluorescence intensity of Zo-1 and Claudin1 was attenuated in the DHSD group but restored in the LWGH group compared to the control group (Figure 6A). In addition using Western blotting to detect colonic tight junction protein expression (Figure 6C,D), compared to the control group, the protein expression of Zo-1, Claudin1, and MUC2 was significantly reduced in the DHSD group (*p* < 0.01) and the levels of these proteins were significantly increased after LWG treatment (*p* < 0.01); similarly, the mRNA levels of *Zo-1*, *Claudin1*, and *MUC2* in each group is consistent with the protein expression (Figure 6B). Further observation of the microstructure of the colon using transmission electron microscopy showed depletion of microvilli and disruption of epithelial junctions in the DHSD group, as well as structural alterations of the mitochondria and nuclei, suggesting that the epithelial cells were damaged, and the LWG alleviated the above mentioned negative effects (Figure 6E).

To verify the authenticity of the network pharmacology results, we verified the protein expression of the STAT3/PI3K/AKT cascade signaling pathway (Figure 6F,G), and the results showed that the phosphorylation levels of STAT3, PI3K, and AKT were significantly increased in the DHSD group, and the phosphorylation levels of the three proteins were reduced after LWG treatment in a dose-dependent manner.

### 3.7. LWG Protects the LPS-Induced Cellular Injury Model and Targets STAT3

**Figure 7:**
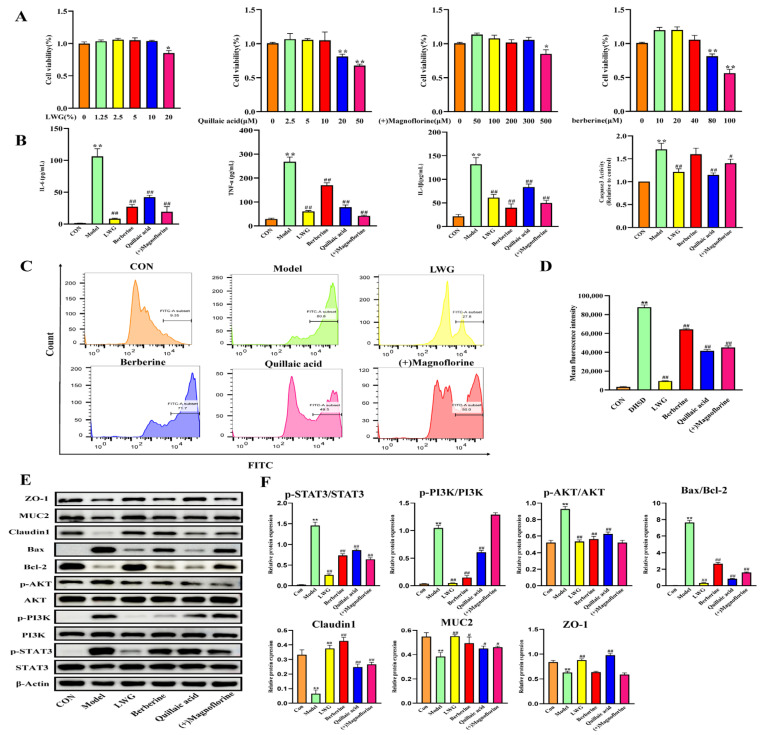
LWG alleviated the LPS-induced IEC-6 cell injury model. (**A**) IEC-6 cell viability assay after 24 h of treatment with different drugs at graded concentrations. (**B**) IL-6, TNF-α, IL-1β, and caspase 3 activities were determined in different groups. (**C**,**D**) The levels of ROS in cells. (**E**,**F**) Protein blot analysis of different subgroups of Bcl-2, Bax, p-STAT3/STAT3, p-AKT/AKT, p-PI3K/PI3K, ZO-1, MUC2, Claudin1. Statistical comparisons were made with the control group, denoted as * *p* < 0.05, ** *p* < 0.01. Comparisons with the DHSD group are denoted as ^#^ *p* < 0.05, ^##^ *p* < 0.01.

To further validate and compare the therapeutic efficacy of the three key ingredients as well as the LWG-containing serum, we first conducted a drug toxicity test to determine the maximum non-toxic therapeutic dose of the drug. CCK-8 results showed that 10% LWG-containing serum, 100 μM quillaic acid, 300 μM (+)magnoflorine, and 40 μM berberine did not exhibit significant pharmacological toxicity (Figure 7A; *p* > 0.05). Further anti-inflammatory results showed that LWG-containing serum and three key compounds significantly reduced LPS-induced inflammatory factor flare-ups (*p* < 0.01), while all drugs except berberine significantly inhibited caspase3 activity (Figure 7B; *p* < 0.01). The results of flow cytometry showed that LWG significantly reversed the LPS-induced increase in intracellular ROS content (*p* < 0.01, Figure 7C,D). The above results reflect the consistency of the ex vivo and in vivo results.

Western blot results showed that LWG-containing serum up-regulated anti-apoptotic (bcl 2) and inhibited pro-apoptotic (Bax) proteins, and significantly decreased the phosphorylation levels of STAT3, PI3K, and AKT (*p* < 0.01), which was consistent with the in vivo results (Figure 7E,F). The expression of tight junction proteins (Zo-1, Claudin1, and MUC2) was significantly decreased (*p* < 0.01) 24 h after LPS induction, and this phenomenon was reversed by LWG-containing serum (Figure 7E,F). Taken together, the therapeutic effect of LWG-containing serum was more satisfactory than that of the other three drugs.

**Figure 8:**
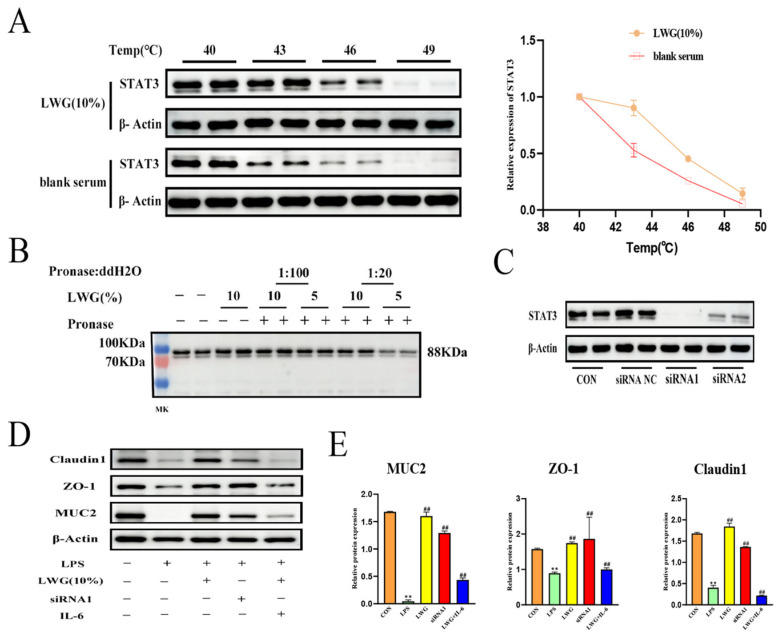
LWG exerts therapeutic effects by targeting STAT3. (**A**) Thermal stability of STAT3 protein determined by CETSA. (**B**) DARTS predicts the interaction between LWG and STAT3. (**C**) Analysis of STAT3 protein expression after specific siRNA treatment. (**D**,**E**) Activation or inhibition of STAT3 on tight junction proteins. Statistical comparisons were made with the control group, denoted as ** *p* < 0.01. Comparisons with the DHSD group are denoted as ^##^ *p* < 0.01.

To verify whether LWG could play a therapeutic role by targeting STAT3, we explored using CETSA and DARTS technology. CETSA results showed that STAT3 proteins in the presence of LWG-containing serum exhibited good stability compared to controls, a result that demonstrates that LWG targets STAT3 (Figure 8A). In addition, we performed DARTS experiments, and similarly, LWG-treated STAT3 became more tolerant to pronase, flanking the binding of LWG to STAT3 (Figure 8B). Finally, the knockdown of STAT3 using small interfering RNA did not significantly reduce tight junction protein expression 24 h after LPS induction (Figure 8D,E). The therapeutic effect of LWG was attenuated after the addition of STAT3 agonist (IL-6100 ng/mL) (Figure 8D,E). In summary, LWG can exert a therapeutic effect in the treatment of DHSD by targeting and binding STAT3.

## 4. Discussion

DHSD is a common functional intestinal disease in the tropics. Poor environmental factors and dietary habits are the main predisposing factors [32], and the increasing incidence and lack of safe and effective therapeutic drugs have increased the economic burden and psychological pressure on patients [33]. In recent years, TCM has made outstanding achievements in the treatment of gastrointestinal diseases, with the advantages of few side effects and low cost [34]. LWG is a traditional Chinese medicine granule with the efficacy of “clearing heat and stopping diarrhea”, which has shown satisfactory effects against DHSD in clinical practice. However, the unknown pharmacodynamic composition and mechanism limit its further development; therefore, this study integrates the methods of serum pharmacochemistry, network pharmacology, and in vivo and ex vivo experiments to reveal the pharmacodynamic composition and the exact mechanism of LWG.

Network pharmacology is a new approach characterized by “network targets” that can effectively predict the regulatory mechanisms of herbal formulations [35]. Although the effects of cyberpharmacology have been widely recognized, it is based on databases for target prediction, and the accuracy and reliability of the findings are still not fully convincing [36]. Thus, combining serum pharmacochemistry can greatly improve the accuracy of study results [37]. By analyzing the mass spectrometry data, we screened a total of 91 compounds for possible therapeutic effects, and there is no doubt that this discovery will deepen the foundation of LWG’s future research. Based on 91 compounds whose targets were further analyzed, we visualized the complex interactions between the targets and DHSD and identified compounds that may play a key role, for example (+) magnoflorine, berberine, and quillaic acid. Meanwhile, STAT3 was found to play a crucial role in the network structure, which was shown in our molecular docking prediction results to exhibit strong affinity with three key compounds, further suggesting that STAT3 may be a key target for LWG to exert its therapeutic effects. The results of DARTS and CETSA experiments verified the above results, confirming the effect of LWG on the STAT3 targeting effect. Finally, the enrichment results showed that PI3K/AKT signaling and apoptosis-associated pathways were considered as a possible mechanism.

We first defined the initial efficacy of LWG using DAI score with weight loss. The results showed that LWG reversed weight loss and significantly reduced DAI scores in DHSD mice, indicating that LWG has a therapeutic effect on DHSD. Previous studies have shown that DHSD is primarily characterized by a burst of pro-inflammatory factors in the colon with significant pathologic damage [11]. H&E staining results confirmed this finding, with DHSD mice displaying severe pathological damage that was significantly ameliorated after LWG intervention. It is well known that goblet cells can secrete MUC2 protein, which is an important component of the colonic barrier [38]. The number of goblet cells was quantified by AB–PAS staining, and the results showed that LWG alleviated cuprocytopenia and protected the integrity of the colonic barrier in DHSD mice. Notably, ex vivo and in vivo results confirmed that LWG up-regulated the protein expression of MUC2, further validating the protective effect of LWG on barrier function. Previous studies have shown that intestinal barrier damage is partially attributable to oxidative damage [39]. According to our experimental results, antioxidant enzyme activities in the intestines of DHSD mice were significantly reduced, demonstrating a potential link between oxidative stress and barrier damage, an adverse effect that was treated by LWG. Finally, ELISA results demonstrated a burst of pro-inflammatory factors in the colon of DHSD mice that could be contained by LWG, particularly IL-6, in keeping with previous studies [40].

STAT3 is involved in cellular physiological events in vivo, including apoptosis, cell proliferation, and cell cycle regulation, and is usually activated by IL-6 [41]. In general, STAT3 is often thought to be associated with cancer as well as immune responses; however, recent studies have shown that STAT3 appears to modulate downstream signaling to stimulate inflammatory bowel disease, such as PI3K/AKT [42]. Our findings corroborate this conjecture, as STAT3 was phosphorylated by excess IL-6 and protein expression of p-PI3K; p-AKT was elevated, as well, leading to up-regulation of the Bax/Bcl-2 ratio and an increase in caspase3 activity, which ultimately stimulated apoptosis at the colon, and the above negative effects were significantly reversed by LWG.

Apoptosis is an early event in intestinal barrier damage [43], and overactive apoptosis is thought to contribute to the disruption of intestinal barrier function [44]. In the present study, the expression of tight junction proteins such as ZO-1, Claudin1, and MUC2 was significantly decreased after modeling, and the intestinal dysfunction induced by the DHSD model was restored after LWG treatment, and the above results were further verified in vitro. Notably, the therapeutic effect of LWG was counteracted after the addition of exogenous IL-6.

Numerous studies have shown that single drugs are far less effective than combinations of multiple drugs [45], and while providing superior therapeutic effects, combinations of multiple drugs reduce the incidence of adverse effects by lowering the individual dose [46]. In this study, although the three key compounds showed relatively good anti-inflammatory and anti-apoptotic effects in vitro, the therapeutic effect of LWG-containing serum was more satisfactory, which side-by-side illustrates the synergistic and holistic nature of the herbal formula particles. In conclusion, our study validated the therapeutic effect of LWG against DHSD and identified the mechanism, providing a potential candidate drug for clinical use against DHSD.

## 5. Conclusions

Our results revealed that LWG inhibits colonic inflammation and oxidative stress, restores intestinal barrier function, and exerts a therapeutic effect on DHSD by inhibiting the IL-6/STAT3/PI3K/AKT signaling pathway and identifying the targeted binding of LWG to STAT3.

## Figures and Tables

**Table 1:** Detailed information on 91 drug prototype components.

NO.	NAME	TPSA	M/W	Rbon	Hacc	Hdon	MLogP
MOL1	N3,N4-Dimethyl-L-arginine	99.74	202.25	7	4	4	−2.52
MOL2	Prolylleucine	95.94	362.42	10	5	2	1.5
MOL3	3-Hydroxysebacic acid	94.83	218.25	9	5	3	0.72
MOL5	Prednisolone	94.83	360.44	2	5	3	1.3
MOL6	Quillaic acid	94.83	486.68	2	5	3	4.04
MOL7	Aloe-emodin	94.83	270.24	1	5	3	0.1
MOL8	Emodin	94.83	270.24	0	5	3	0.36
MOL9	N-Formylmethionine	91.7	177.22	6	3	2	0.25
MOL11	Telocinobufagin	90.9	402.53	1	5	3	2.75
MOL13	Xanthurenic acid	90.39	205.17	1	4	3	−0.15
MOL20	4-Hydroxyhippuric acid	86.63	195.17	4	4	3	0.28
MOL23	DL-Tryptophan	79.11	204.23	3	3	3	−1.66
MOL25	Deoxycholic acid	77.76	392.57	4	4	3	3.88
MOL29	2-Mercaptobenzothiazole	76.12	167.25	0	0	1	1.42
MOL30	Sakuranetin	75.99	286.28	2	5	2	0.96
MOL32	3-tert-Butyladipic acid	74.6	202.25	6	4	2	1.55
MOL36	Itaconic acid	74.6	130.1	3	4	2	−0.23
MOL37	Terephthalic acid	74.6	166.13	2	4	2	1.2
MOL40	Dantron	74.6	240.21	0	4	2	0.67
MOL41	Rubiadin	74.6	254.24	0	4	2	0.92
MOL42	5-Hydroxyindole-3-acetic acid	73.32	191.18	2	3	3	0.53
MOL44	Bufalin	70.67	386.52	1	4	2	3.58
MOL48	N8-Acetylspermidine	67.15	187.28	9	3	3	0.14
MOL49	Senkyunolide H	66.76	224.25	2	4	2	0.83
MOL50	Isovanillic acid	66.76	168.15	2	4	2	0.74
MOL51	5-Methoxysalicylic acid	66.76	168.15	2	4	2	0.74
MOL53	Hexanoylglycine	66.4	173.21	7	3	2	0.53
MOL55	Cinnamoylglycine	66.4	205.21	5	3	2	1.08
MOL57	3-Methylhippuric acid	66.4	193.2	4	3	2	1.14
MOL58	N-Tigloylglycine	66.4	157.17	4	3	2	0.1
MOL59	4-Methylhippuric acid	66.4	193.2	4	3	2	1.14
MOL62	Uracil	65.72	112.09	0	2	2	−0.8
MOL63	Xylenesulfonate	65.58	185.22	1	3	0	1.78
MOL65	Spermidine	64.07	145.25	7	3	3	0.08
MOL67	Monobutyl phthalate	63.6	222.24	6	4	1	2.39
MOL68	2-Furyl(5-hydroxy-1-Benzofuran-3-yl)methanone	63.58	228.2	2	4	1	0.37
MOL69	Levetiracetam	63.4	170.21	3	2	1	−0.27
MOL71	L-Isoleucine	63.32	131.17	3	3	2	−1.82
MOL79	Estriol	60.69	288.38	0	3	3	2.65
MOL80	(+)-Magnoflorine	58.92	342.41	2	4	2	−1.71
MOL81	Creatinine	58.69	113.12	0	2	1	−0.53
MOL82	3-Hydroxydecanoic acid	57.53	188.26	8	3	2	1.7
MOL85	3-Hydroxybutyric acid	57.53	104.1	2	3	2	−0.39
MOL87	4-Hydroxybenzoic acid	57.53	138.12	1	3	2	0.99
MOL88	Hecogenin	55.76	430.62	0	4	1	4.09
MOL89	Dodecamethylcyclohexasiloxane	55.38	444.92	0	6	0	−1.28
MOL95	Melatonin	54.12	232.28	5	2	2	1.86
MOL96	Indole-3-carboxylic acid	53.09	161.16	1	2	2	1.08
MOL97	Dibutyl phthalate	52.6	278.34	10	4	0	3.43
MOL100	5-Phenylnicotinic acid	50.19	199.21	2	3	1	0.5
MOL103	Cyclo(leucylprolyl)	49.41	210.27	2	2	1	0.64
MOL104	N-Acetyltyramine	49.33	179.22	4	2	2	1.27
MOL113	3,5-Dimethyl-4- Methoxybenzoic acid	46.53	180.2	2	3	1	1.94
MOL114	Vanillin	46.53	152.15	2	3	1	0.51
MOL115	N,N′-Dicyclohexylurea	46.33	224.34	3	1	1	2.56
MOL117	2-Methyl-6-phenylpyrimidin-4-ol	45.75	186.21	1	2	1	1.46
MOL118	Primobolan	43.37	344.49	2	3	0	4.1
MOL121	Berberine	40.8	336.36	2	4	0	2.19
MOL122	Fraxinellone	39.44	232.28	1	3	0	2.08
MOL123	7-Methoxy-4-methylcoumarin	39.44	190.2	1	3	0	1.63
MOL125	Parthenolide	38.83	248.32	0	3	0	2.47
MOL126	Arglabin	38.83	246.3	0	3	0	2.47
MOL127	4-Propylbenzoic acid	37.3	164.2	3	2	1	2.55
MOL128	Altrenogest	37.3	310.43	2	2	1	3.77
MOL129	2-Norbornaneacetic acid	37.3	154.21	2	2	1	1.88
MOL130	Cyclopentylacetic acid	37.3	128.17	2	2	1	1.23
MOL132	Trichloroacetic acid	37.3	163.39	1	2	1	0.89
MOL133	2-Methylbenzoic acid	37.3	136.15	1	2	1	−2.16
MOL134	Benzoic acid	37.3	122.12	1	2	1	1.6
MOL135	Nandrolone	37.3	274.4	0	2	1	3.36
MOL138	4′-(Imidazol-1-yl)acetophenone	34.89	186.21	2	2	0	0.96
MOL139	2,6-Di-tert-butyl-1,4-benzoquinone	34.14	220.31	2	2	0	2.19
MOL140	Phthaldialdehyde	34.14	134.13	2	2	0	0.77
MOL141	Progesterone	34.14	314.46	1	2	0	3.95
MOL142	3,3′,5,5′-Tetramethyldiphenoquinone	34.14	240.3	0	2	0	2.45
MOL144	Perillartine	32.59	165.23	2	2	1	1.98
MOL145	Curcumol	29.46	236.35	1	2	1	3.15
MOL147	N,N-Dimethyldecylamine N-oxide	29.43	201.35	9	1	0	1.46
MOL148	2-Oxindole	29.1	133.15	0	1	1	1.13
MOL150	Norharman	28.68	168.19	0	1	1	1.62
MOL151	Acetyl-β-methylcholine	26.3	195.69	4	2	0	−1.67
MOL152	Nabumetone	26.3	228.29	4	2	0	2.9
MOL153	Isobornyl methacrylate	26.3	222.32	3	2	0	3.19
MOL155	Isoalantolactone	26.3	232.32	0	2	0	3.35
MOL156	Dehydrocostus lactone	26.3	230.3	0	2	0	3.26
MOL157	Clareolide	26.3	250.38	0	2	0	3.8
MOL163	Spiroxamine	21.7	297.48	6	3	0	3.14
MOL166	Acetophenone	17.07	120.15	1	1	0	1.78
MOL167	2,4-Dimethylbenzaldehyde	17.07	134.18	1	1	0	2.1
MOL168	Diphenylamine	12.03	169.22	2	0	1	3.34
MOL169	Tributylamine	3.24	185.35	9	1	0	3.41

Hdon: hydrogen bond donors, Hacc: hydrogen bond acceptors, Rbon: the number of rotatable bonds, MLOGP: the lipid–water partition coefficient, M/W: the relative molecular mass, TPSA: topological polar surface area.

## Data Availability

The original contributions presented in the study are included in the article/Supplementary Material, further inquiries can be directed to the corresponding author.

## Supplementary Materials

The following supporting information can be downloaded at https://www.mdpi.com/article/10.3390/antiox13060661/s1, Figure S1. HPLC chromatogram of LWG. Figure S2. Total ion chromatograms of compounds in rat serum obtained by UPLC-MS/MS. Table S1. Evaluation of disease activity index (DAI). Table S2. Primers and probes for real-time qRT-PCR. Table S3. Important parameters during Western blot experiments.

## Abbreviations

AB-PAS	Alcian blue-periodic acid-Schiff
BBR	Berberine
BCA	Bicinchoninic Acid Assay
BP	Biological process
BTW	Pulsatilliae radix
CAT	Catalase
CC	Cellular component
CCK-8	Cell Counting Kit-8
CETSA	Cellular thermal shift assay
DAI	Disease activity index
DAPI	4′,6-diamidino-2-phenylindole
DARTS	Drug affinity responsive target stability
DCFH-DA	2′,7′-Dichlorodihydrofluorescein diacetate
DHSD	Dampness-heat syndrome diarrhea
DHT	Rhubarb charcoal
FITC	Fluorescein 5-isothiocyanate
ECL	Enhanced chemiluminescence
GO	Gene Ontology
Hacc	Hydrogen bond acceptors
Hdon	Hydrogen bond donors
H&E	Hematoxylin eosin
HL	Coptidis rhizoma
KEGG	Kyoto Encyclopedia of Genes and Genomes
LGC	Geranium wilfordii maxim
LWG	Lian weng granules
MCC	Maximum cluster centrality
MF	Molecular function
MLOGP	The lipid–water partition coefficient
M/W	The relative molecular mass
OMIM	Online Mendelian Inheritance in Man
PBS	Phosphate buffer saline
PBST	Phosphate buffered saline with tween 20
PI3K	Phosphoinositide 3-Kinase
PPI	Protein-protein interaction network
Rbon	The number of rotatable bonds
SDS-PAGE	Sodium dodecyl sulfate–polyacrylamide gel electrophoresis
SiSTAT3	STAT3-specific small interfering RNA
STAT3	Signal transducer and activator of transcription-3
SOD	Superoxide dismutase
TCM	Traditional Chinese medicine
TEM	Transmission electron microscopy
TTD	Therapeutic target database
TUNEL	TdT-mediated dUTP-biotin nick end labeling
TPSA	Topological polar surface area
UHPLC-MS/MS	Ultra-high performance liquid chromatography-tandem mass spectrometry
ZO-1	Tight junction protein ZO-1.
