# Supplementary material for: Lianweng Granules Alleviate Intestinal Barrier Damage via the IL-6/STAT3/PI3K/AKT Signaling Pathway with Dampness-Heat Syndrome Diarrhea
Source: Antioxidants (Basel). 2024 May 28;13(6):661. doi: 10.3390/antiox13060661 (PMC11201218; doi:10.3390/antiox13060661)
Supplement: Supplementary file 1 [file antioxidants-13-00661-s001.zip › antioxidants-3029176-supplementary.pdf]

1. Quality identification of LWG by HPLC

Accurately weighed berberine hydrochloride was used as the reference solution and methanol was added to make a solution containing 40 μg/mL. An accurately weighed sample of 0.15g LWG powder was diluted with 50mL of methanol-hydrochloric acid (100:1) and the weight was recorded. Heat reflux for 30 min and make up the lost weight with solvent. Before injection, all samples were filtered through a 0.45 μm membrane filter. The standard of this assay required that LWG contains no less than 10.5 mg of berberine (BBR) pergram.

Chromatographic separation was performed on a Waters 1525 HPLC system (Waters Corp., Milford, Massachusetts, USA) equipped with a VP ODS C18 column (250 mm × 4.6 mm, 5 μm). Samples were eluted using a mixture of solvent A (acetonitrile) and solvent B (0.3% phosphoric acid solution) at a ratio of 32:68. The flow rate was 1.0 mL/min, the column temperature was 25 °C, the detection wavelength was 348 nm, and the injection volume was 10 μL.

Figure S1. HPLC chromatogram of LWG

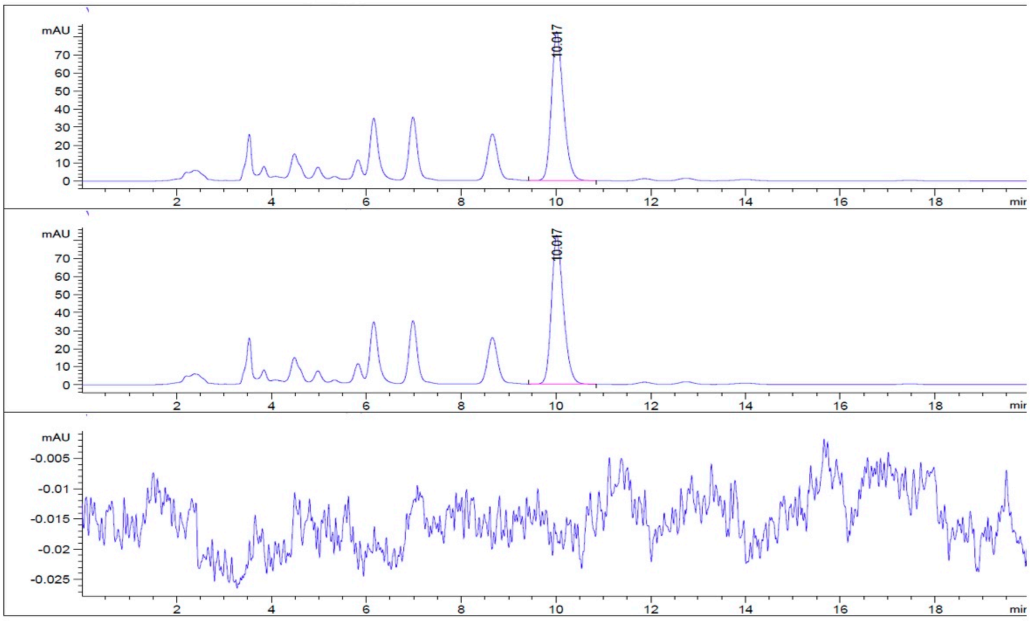

2. MS analysis conditions

The samples of 10 mg were dissolved in 1 mL 80% methanol. After sonicated for 10 min, and

centrifuged at 14,000 rpm for 10 min, a 500 μL of the supernatant was passed through a 0.22 μm filter membrane and then transferred to UHPLC-MS/MS analysis. Separation was performed using the ACQUITY UPLC HSS T3 (2.1×100 mm, 1.8 μm). Elution was carried out at a stream rate of 0.3 mL/min utilizing 0.1% formic acid in water (A) and 0.1% formic acid in acetonitrile (B).

A Q Exactive Focus mass spectrometer coupled with Xcalibur software was employed to obtain the MS and MS/MS data with the IDA acquisition mode. Mass spectra were recorded using the Full MS-ddMS2 mode, simultaneous in both positive and negative modes. During each acquisition cycle, the mass range was from 100 to 1,200. Capillary temp:320℃, Aux gas heater temp:350℃, Sheath gas flow rate: 40 L/min, Aux gas flow rate: 15 L/min, ion source voltage: 3.2 kV, Full MS resolution: 70000, MS/MS resolution: 17500, Collision energy: 30/40/50 in NCE mode. Mass spectra were imported raw using Compound Discoverer 3.3 software. Materials identification of peaks containing MSMS data was performed using the secondary mass spectrometry database (Tsinghua University) and Mzcloudthe database.

3. Primer sequences for SiSTAT3

5-3:CCUGAGUUGAAUUAUCAGCUU
3-5:GGACUCAUCUUAAUAGUCGAA

4. Table S1. Evaluation of disease activity index (DAI)

Score	Weight loss(%)	Stool consistency	Hematochezia
0	none	normal	no hemafecia
1	0-5	loose stools	stool occult blood
2	5-10	loose stools	stool occult blood
3	10-15	loose stools	bloody stools
4	> 15	loose stools	bloody stools

5. Table S2. Primers and probes for real-time qRT-PCR

Gene	Left primer	Right primer	References

Gene	Left primer	Right primer	References
IL-6	TCCAGTTGCCTTCTTGGGAC	GTGTAATTAAGCCTCCGACTTG	[24]
TNF-α	CACGTCGTAGCAAACCACCAAGTGGA	TGGGAGTAGACAAGGTACAACCC	[25]
IL-1β	TGCCACCTTTTGACAGTGATG	AAGGTCCACGGGAAAGACAC	[26]
Bax	GGCCCACCAGCTCTGAGCAGA	GCCACGTGGGCGTCCCAAAGT	[27]
Bcl2	GTGGAGGAGCTCTTCAGGGA	AGGCACCCAGGGTGATGCAA	[27]
MUC2	ACCTACCATCCTATGAGCGAG	GGTTTGTGTAAGAGAGGCTGC	[28]
Claudin1	ATGCAAAGATGTTTTGCCAC	TACAAATTCCCATTGCAGCCC	[28]
Zo-1	TCAGAGCCCTCCGATCATTC	GCTTTGGGTGGATGATCGTC	[29]
β-Actin	TCCTCCTGAGCGCAAGTACTCC	CATACTCCTGCTTGCTGATCCAC	[30]

6. Figure S2. Total ion chromatograms of compounds in rat serum obtained by UPLC-MS/MS

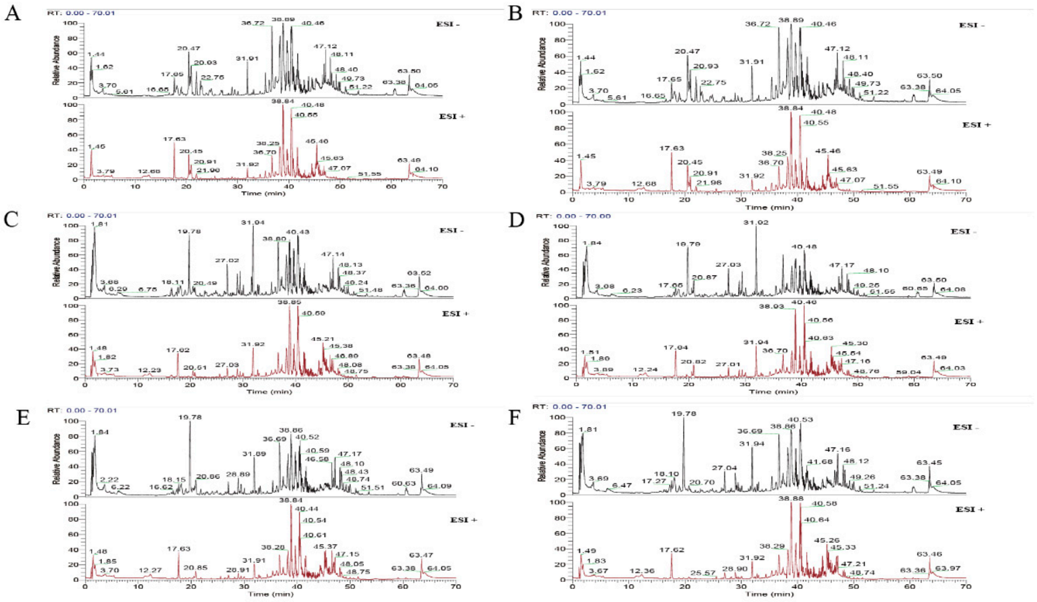

(A) control group. (B) LWG group. (C). BTW group. (D). DHT group. (E) HL group. (F) LGC group. (top: negative ion mode, bottom: positive ion mode).

7. Table S3. Important parameters during Western blot experiments

Name	Provider	Catalog Number	dilution	Blocking Buffer
TNF-α	abcam	ab183218	1:2000	Skimmed milk
IL-6	abcam	ab290735	1:1000	Skimmed milk
IL-1β	abcam	ab283818	1:1000	Skimmed milk
Bax	Proteintech	50599-2-Ig	1:6000	Skimmed milk
Bcl-2	Proteintech	68103-1-Ig	1:20000	Skimmed milk
ZO-1	Proteintech	21773-1-AP	1:10000	Skimmed milk
MUC-2	Proteintech	27675-1-AP	1:1000	Skimmed milk
AKT	Proteintech	60203-2-Ig	1:10000	Skimmed milk
p-AKT	Proteintech	66444-1-Ig	1:15000	5%BSA
PI3K	Abmart	T40115	1:2000	Skimmed milk
p-PI3K	Abmart	T40116	1:1000	5%BSA
STAT3	HuaBio	ET1605-45	1:1000	Skimmed milk
p-STAT3	Abmart	T56566	1:1000	5%BSA
β-Actin	Proteintech	66009-1-Ig	1:50000	Skimmed milk
